# DNA methylation in blood cells is associated with cortisol levels in offspring of mothers who had prenatal post‐traumatic stress disorder

**DOI:** 10.1002/smi.3131

**Published:** 2022-02-10

**Authors:** Peter Daniel Fransquet, Line Hjort, Feride Rushiti, Shr‐Jie Wang, Sebahate Pacolli Krasniqi, Selvi Izeti Çarkaxhiu, Dafina Arifaj, Vjosa Devaja Xhemaili, Mimoza Salihu, Nazmie Abullahu Leku, Joanne Ryan

**Affiliations:** ^1^ School of Public Health and Preventive Medicine Biological Neuropsychiatry and Dementia Unit Monash University Melbourne Victoria Australia; ^2^ Department of Obstetrics Center for Pregnant Women with Diabetes Rigshospitalet Copenhagen Denmark; ^3^ Novo Nordisk Foundation Center for Basic Metabolic Research Metabolic Epigenetics Group Faculty of Health and Medical Sciences University of Copenhagen Copenhagen Denmark; ^4^ Kosovo Rehabilitation Center for Torture Victims Pristina Albania; ^5^ Danish Institute Against Torture (DIGNITY) Copenhagen Denmark

**Keywords:** *BDNF*, cortisol, *CRH*, *CRHR1/2*, DNA methylation, epigenetics, *FKBP5*, intergenerational, maternal PTSD, *NR3C1/2*, offspring, war

## Abstract

Maternal stress during pregnancy is associated with differential DNA methylation in offspring and disrupted cortisol secretion. This study aimed to determine methylation signatures of cortisol levels in children, and whether associations differ based on maternal post‐traumatic stress disorder (PTSD). Blood epigenome‐wide methylation and fasting cortisol levels were measured in 118 offspring of mothers recruited from the Kosovo Rehabilitation Centre for Torture Victims. Mothers underwent clinically administered assessment for PTSD using Diagnostic and Statistical Manual of Mental Disorders. Correlations between offspring methylation and cortisol levels were examined using epigenome‐wide analysis, adjusting for covariates. Subsequent analysis focussed on a priori selected genes involved in the hypothalamic–pituitary–adrenal (HPA) axis stress signalling. Methylation at four sites were correlated with cortisol levels (cg15321696, *r* = −0.33, cg18105800, *r* = +0.33, cg00986889, *r* = −0.25, and cg15920527, *r* = −0.27). In adjusted multivariable regression, when stratifying based on prenatal PTSD status, significant associations were only found for children born to mothers with prenatal PTSD (*p* < 0.001). Several sites within HPA axis genes were also associated with cortisol levels in the maternal PTSD group specifically. There is evidence that methylation is associated with cortisol levels, particularly in offspring born to mothers with prenatal PTSD. However, larger studies need to be carried out to independently validate these findings.

## 1 INTRODUCTION

Stress related disorders during pregnancy, such as high stress, depression and anxiety have been shown to affect foetal development and lead to a multitude of poor birth and later health outcomes (Glover, 2014). This includes low birthweight for gestational age, negative effects on brain and cognitive development, an increased likelihood of social and behavioural problems, and a higher risk of stress‐related mood disorders in childhood which can persist into later life (Jarde et al., 2016). Maternal stress during pregnancy may disrupt the setting of offspring hypothalamic–pituitary–adrenal (HPA) axis signalling, resulting in aberrant cortisol secretion (Castelli et al., 2020). Epigenetic mechanisms, including DNA methylation, are likely to play an important role, and could help explain the lasting effects of early‐life maternal stress on the offspring (J. Ryan et al., 2017).

Cortisol is a glucocorticoid hormone secreted from the *zona fasciculata* of the adrenal cortex when stimulated by adrenocorticotropin release from the pituitary gland (Lightman et al., 2020), and is primarily secreted in response to stress (Pulopulos et al., 2020). Epigenetic mechanisms, such as DNA methylation, play a role in cortisol (HPA‐axis) signalling. DNA methylation is involved in cortisol production (Kometani et al., 2017), and glucocorticoid receptor activity (Watkeys et al., 2018). Further, DNA methylation has been associated with cortisol levels (Wrigglesworth et al., 2019) and shown to mediate the association between childhood trauma and cortisol stress reactivity (Argentieri et al., 2017; Houtepen et al., 2016).

Post‐traumatic stress disorder (PTSD) is characterised by a re‐experiencing of traumatic events, associated with symptoms including intrusive thoughts and memories, active avoidance, negative changes to mood and cognition, and changes to reactivity and arousal (Miao et al., 2018). Post‐traumatic stress disorder is thought to have negative intergenerational effects, passed on from mothers to offspring prenatally (Miao et al., 2018; von der Warth et al., 2020; Yehuda & Bierer, 2007), with biological mechanisms such as DNA methylation likely to be involved (J. Ryan et al., 2016). One of the primary biological characteristics of PTSD is disrupted cortisol secretion (Speer et al., 2019). Differences in cortisol, and cortisol signalling have also been observed in the offspring of mothers that have experienced prenatal PTSD (Bader et al., 2014; Liu et al., 2016), which may be associated with poor psychiatric outcomes. We have recently demonstrated differential blood DNA methylation profiles in offspring of mothers who had prenatal PTSD compared to those without (Hjort et al., 2021). This is also supported by previous studies showing that intergenerational effects are partly attributed to epigenetic processes (Perroud et al., 2014; Youssef et al., 2018). The mechanisms of this transgenerational effect of PTSD in pregnancy could be due to differential DNA methylation which is associated with cortisol levels in offspring.

The aim of this study was to identify DNA methylation signatures associated with fasting cortisol levels in children, at the epigenome‐wide level and then focus on specific candidate genes of the HPA stress axis. A secondary aim was to ascertain whether maternal PTSD during pregnancy modifies any observed associations.

## 2 METHODS

### 2.1 Study cohort

This study involved women recruited from the Kosovo Rehabilitation Centre for Torture Victims (KRCT) and their youngest offspring. Participant characteristics have been described in detail previously (Hjort et al., 2021). The KRCT recruited 130 women aged between 30 and 59 years, who had experienced torture and/or sexual violence during the Kosovo war. Participants had given birth to at least one child after the war, which was not related to sexual assault. All women were of Albanian ethnicity, born in Kosovo, and had a home address in Kosovo during the war in 1999. Clinical assessments and questionnaires were conducted during 2019 by psychologists and medical doctors at KRCT for all participants. Diagnosis of PTSD was based on the Diagnostic and Statistical Manual of Mental Disorders criteria's “Clinician‐Administered PTSD Scale” (CAP‐IV) (American Psychiatric Publishing Inc., 1994), which had been translated and validated in Albanian language (Turner et al., 2003). Socio‐demographic and lifestyle data were also collected. These included age, educational attainment (none, primary, secondary, or higher), marital status (married, divorced, single, widowed), place of residence (city or village) and prenatal cigarette smoking.

### 2.2 Ethics statement

This study was approved by the commission for the ethical issues within Kosovo doctor's chamber. The study was carried out in accordance with the Ministry of Health Central Ethics Committees in Kosovo, as per Kosovar Government guidelines, and with the Helsinki Declaration. All participants who agreed to take part provided informed consent. They were informed that they have the right to withdraw from the study at any time. Any participant suffering from adverse effects of trauma was referred to a psychologist or medical doctor at KRCT. The information provided by the study participants was treated throughout the process with confidentiality according to the Kosovar law and Declaration of Helsinki II on biomedical research and complied with general data protection regulation.

### 2.3 Blood collection

Fasting blood samples were collected from 120 of the youngest offspring born to each woman, by lab technicians at the Tirana Laboratory, Pristina, Kosovo in March, and April of 2019. After a 20‐min rest period in a comfortable environment, a sample was collected from each child between 7:30 and 9:30am in an 6 ml tube (SARSTEDT AG & Co.). Cortisol was measured in offspring blood samples using electrochemical luminescence immunoassay and reported in International System of Units (nmol/L) (COBAS E411, Roche). The reference range for cortisol levels in the laboratory in Pristina (Kosovo) was used to identify low and high cortisol levels. A separate sample for DNA extraction, was collected in an 6 ml EDTA plasma tube and stored at −20°C for 2–3 weeks, before being shipped to Denmark where it was stored at −80°C until processed (DNeasy DNA blood kit, Qiagen).

### 2.4 DNA methylation profiling and bioinformatics

Epigenome‐wide DNA methylation data was generated using the Illumina's Infinium HumanMethylationEPIC BeadChip (Illumina), processed by GenomeScan in Leiden, Netherlands. After removing one sibling from two sets of twin pairs, methylation data of 118 offspring were available for analyses.

Pre‐processing of data was carried out using R version 4.0.3 (R Core Team, 2021), and the *minfi* package (Aryee et al., 2014). Probes at methylation sites (also known as cytosine‐phosphate‐guanine dinucleotides or CpGs) where array signals were not discernible from background noise (at *P* > 0.01) were removed from the data set using the ‘*detectionP’* function of *minfi*. No samples required removal as after removing problematic probes, no sample was missing data, and all were uniformly bi‐modally distributed. Child biological sex was determined and confirmed using the *‘getSex’* function of *minfi*. Data were normalized using the subset quantile normalisation method (Wu & Aryee, 2010). After removing sex chromosome probes, known cross‐reactive probes (Pidsley et al., 2016), and probes containing a single nucleotide polymorphism at the methylation site (CpG) or within a single‐base extension (SBE) (Supplementary Table 1), 625,431 CpGs were available for analysis.

Cytosine‐phosphate‐guanine dinucleotides methylation signal intensities were then transformed into M‐values for analysis (log2 unmethylated/methylated signal intensity), and *β*‐values for biological interpretation (methylation between 0 and 1 at each site). M‐values are preferred for statistical analysis due to their bi‐modal distribution, which reflects patterns of methylation across the epigenome (Du et al., 2010). Blood cell estimation was carried out, using the *‘estimateCellcounts2’* function of the *FlowSorted.Blood.EPIC* package (Salas & Koestler, 2018). This function estimates the proportions of B cells (CD19+), T lymphocytes (CD4+ and CD8+), monocytes (CD14+), neutrophils and natural killer cells (CD56+) in blood. As neutrophils were the most prominent cell proportion (mean = 49.9%, SD = 0.08), this estimate was left out of adjustment models.

### 2.5 Statistical analysis: Epigenome wide association study

To identify differentially methylated CpGs associated with cortisol levels, two separate analyses were carried out. One to find associations between methylation and cortisol as a continuous measure (ranged between 10.7 and 722.8 nmol/L), and another using categorical measures to observe if methylation differs between groups of low (≤170 nmol/L) or high (≥550 nmol/L) cortisol compared to normal (>171 to <549 nmol/L). The *cate* package, which removes unwanted variation while controlling for known variables in modelling, was used to carry out high dimensional factor analysis and confounder adjusted multiple testing (Wang & Zhao, 2020). Models assessed continuous and categorical cortisol levels associated with differential methylation, and adjusted for the child's age and sex, the mothers age, level of education, marital status, living location, and pregnancy smoking status, as well as EPIC array chip number for batch effect, and estimated cell proportions (not including neutrophils). A small number of participants were missing data for some of the covariates, and these were imputed using the median value. This included maternal age (*n* = 1), living area (*n* = 1), maternal education (*n* = 3), maternal marital status (*n* = 2) and maternal smoking during pregnancy (*n* = 2). All *p*‐values were adjusted for multiple testing using the Benjamini‐Hochberg method (BH.Adj.P) (Chen et al., 2017). For stratification analysis, we analysed *β*‐values from CpGs with *p* < 0.15 after adjustment for multiple testing. These analyses were conducted using STATA version 14 (StataCorp, 2015).

### 2.6 Candidate gene analysis

Key genes involved in HPA axis signalling for investigation in this study included those which encode signalling molecules such as *brain derived neurotrophic factor* (*BDNF*) (de Assis & Gasanov, 2019), and *corticotropin releasing hormone* (*CRH*) (Zhou & Fang, 2018), as well as glucocorticoid receptors and chaperones involved in receptor activity, *nuclear receptor subfamily 3 group C member 1 and 2 (NR3C1/2*) (Iftimovici et al., 2020; Plieger et al., 2018) and *FK506‐binding protein 51* (*FKBP5/FKBP51*) (Zannas et al., 2016), and corticotropin receptors *CRH receptor 1* and *2* (*CRHR1/2*) (Grimm et al., 2017; Sanabrais‐Jiménez et al., 2019). Methylation data were extracted for each of these genes from the epigenome wide association study (EWAS) data set. Genomic positions of each gene were selected by using the Homo sapiens (human) genome assembly GRCh37 (hg19) reference in the University of California, Santa Cruz genome browser (Haeussler et al., 2019). Genomic regions of probe extraction included the gene body, as well as approximately 25% of the gene size up and down stream. This was done to ensure capturing data from any nearby CpG islands concentrated areas of CpGs, mostly present in gene promotor regions (Hughes et al., 2020), and to capture CpGs surrounding the gene. Correlations between continuous cortisol measures and CpG methylation were carried out using Pearson (normally distributed methylation) and Spearman (non‐normally distributed methylation) methods, and adjusted for multiple comparisons using the Holm method (H.adj.p) (Aickin & Gensler, 1996) in R. STATA was then used for multivariate linear regression for CpGs found to be significantly correlated with cortisol levels using the aforementioned variables, both on the whole sample population and stratified by prenatal PTSD status.

## 3 RESULTS

### 3.1 Participant characteristics

Participant characteristics, stratified by cortisol level, can be seen in Table 1. Just over three quarters of offspring had a normal fasting cortisol level (*n* = 90, 76%). Offspring of mothers with prenatal PTSD had higher cortisol levels. Offspring cortisol levels were also associated with prenatal maternal smoking, living location and maternal marital status.

**TABLE 1:** Study cohort characteristics, according to cortisol levels

	Low cortisol, (≤170 nmol/L)	Normal cortisol, (>171 to <549 nmol/L)	High cortisol, (≥550 nmol/L)	P
Child characteristics				
(*n*, %)	14 (12%)	90 (76%)	14 (12%)	
Cortisol nmol/L, mean (SD)	122.4 (39.9)	329.5 (97.6)	620.9 (53.5)	<0.001***
Age, mean (SD)	9.9 (5.4)	11.9 (5.1)	12.4 (5.9)	0.4
Female sex, *n* (%)	9 (64%)	40 (44%)	4 (29%)	0.2
Maternal characteristics				
Prenatal PTSD, *n* (%)	7 (50%)	65 (72%)	13 (93%)	0.04*
Prenatal smoking, *n* (%)	1 (7%)	33 (37%)	2 (14%)	0.03*
Age, mean (SD)	42.1 (7.5)	42.8 (5.6)	45.6 (5.5)	0.2
Secondary education and above, *n* (%)	5 (36%)	20 (22%)	4 (29%)	0.5
City dwelling, *n* (%)	12 (86%)	59 (66%)	5 (36%)	0.02*
Marital status, *n* (%)	11 (78%)	83 (92%)	9 (64%)	0.008**

Abbreviations: nmol/L, nanomoles per litre; PTSD, post‐traumatic stress disorder; SD, standard deviation.

* *p* < 0.05, ** *p* < 0.01, *** *p* < 0.001.

### 3.2 Epigenome‐wide analysis

**FIGURE 1:**
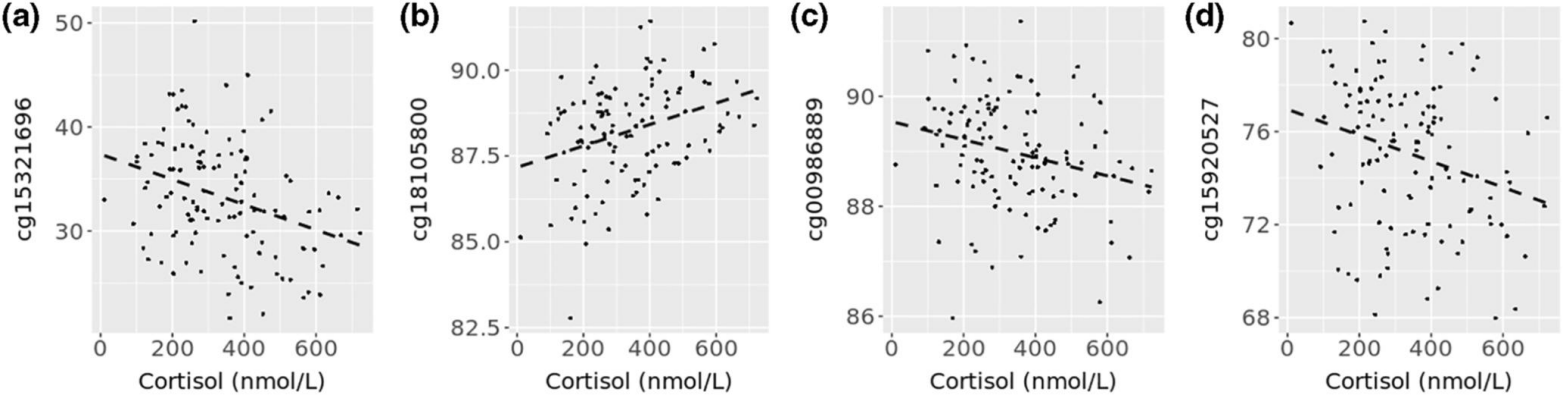
CpGs associated with cortisol levels in epigenome‐wide analysis. Plots (a) to (d) show correlation between methylation and continuous levels of cortisol. (a) cg15321696 (*r* = −0.33, *p* = 0.0002, Benjamini‐Hochberg (BH) Adj.*p* = 0.078), (b) cg18105800 (*r* = 0.33, *p* = 0.0003, BH. Adj.*p* = 0.11), (c) cg00986889 (*r* = −0.25, *p* = 0.006, BH. Adj.*p* = 0.11), (d) cg15920527 (*r* = −0.27, *p* = 0.003, BH. Adj.*p* = 0.11). BH. Adj.p = *p* value after adjustment for estimated blood cell proportions, child age and sex, maternal age, education, marital status, location and prenatal smoking status, batch effects, and multiple testing with a cut off of *p* < 0.15. P = preadjusted significance of correlation/association. R = preadjusted correlation coefficient. *Y*‐axis represents % methylation

In epigenome wide association analysis between methylation and cortisol levels, no CpGs reached adjusted 5% significance levels after correction for multiple testing and controlling for estimated blood cell proportions, child's age and sex, maternal age, education, marital status, living location and prenatal smoking status. Methylation at four CpGs were nominally associated with continuous cortisol levels after adjustments for covariates (*p* < 1 × 10^−6^) and were the most significant sites associated with cortisol after correction for multiple testing (at BH adjusted *p*‐value <0.15) (Table 2). The correlation between methylation and cortisol levels at these CpGs can be seen in Figure 1 (a–d). The strongest effect sizes observed were for CpGs cg15321696 and cg18105800, where the correlation coefficients were −0.33 and + 0.33 respectively. None of these sites were replicated in analysis of categorical cortisol groups, however six separate CpGs were nominally associated with categorical levels of cortisol after aforementioned adjustments (Supplementary Table S2, Supplementary Figure S1).

**TABLE 2:** Top differentially methylated CpGs associated with cortisol levels

				Cate model^b^		
CpG	Location (hg19)	Gene	r	*β* coef.	P	adj.p
cg15321696	chr10:14424332	*FRMD4A*	−0.33	−0.001	1.25 × 10^−07^	0.078
cg18105800	chr3:33420182	*FBXL2*	+0.33	+0.0006	6.88 × 10^−07^	0.11
cg00986889	chr12:6697033	*CHD4*	−0.25	+0.0004	6.67 × 10^−07^	0.11
cg15920527	chr3:14648593	^a^ *CCDC174*	−0.27	−0.0007	5.35 × 10^−07^	0.11

Abbreviations: adj.p = multiple testing adjusted *p*‐value; *β* Coef., Regression coefficient; *CCDC174*, Coiled‐Coil Domain Containing 174; *CHD4*, Chromodomain Helicase DNA Binding Protein 4; CpG, cytosine‐phosphate‐guanine; *FBXL2*, F‐Box And Leucine Rich Repeat Protein 2; *FRMD4A*, FERM Domain Containing 4A; hg19, Homo sapiens (human) genome assembly GRCh37; *r*, correlation coefficient.

^a^ in Gene category denotes that probe is upstream from the gene body/transcription site.

^b^ using m values, adjusted for cell type, child age and sex, maternal age, maternal education, maternal marital status, maternal living area, prenatal smoking, batch, and BH method for multiple testing.

### 3.3 Stratification of multivariate linear regression by prenatal maternal post‐traumatic stress disorder status

Eighty‐five of the 118 mothers had PTSD during pregnancy. For the four CpGs found in epigenome wide association analysis, multivariate linear regression analysis was then stratified according to prenatal maternal PTSD (*n* = 85) or no‐PTSD (*n* = 33). In offspring born to women with maternal PTSD, these CpGs were significantly associated with cortisol levels (Table 3). In contrast, none of these CpGs were associated with cortisol levels in offspring of women without maternal PTSD. Findings concerning the categorical cortisol analysis (Supplementary File, Table S2) were similar, in being only significant for offspring born to mothers with maternal PTSD.

**TABLE 3:** Top CpGs stratified according to maternal post‐traumatic stress disorder during pregnancy

Stratified multivariate linear regression		
(*β* coef. 95%CI, adj.p^a^)		
CpG	No prenatal PTSD (*n* = 33)	Prenatal PTSD (*n* = 85)
cg15321696	0.008, −0.06–0.07, 0.8	−0.018, −0.03 to −0.01, <0.001***
cg18105800	0.004, −0.01–0.02, 0.7	0.004, 0.002–0.006, <0.001***
cg00986889	−0.0002, −0.005 to 0.001, 0.2	−0.003, −0.004 to −0.001, <0.001***
cg15920527	−0.03, −0.07 to 0.01, 0.1	−0.01, −0.014 to −0.005, <0.001***

Abbreviations: *β* Coef., Regression coefficient; CI, confidence interval; CpG, cytosine‐phosphate‐guanine; PTSD, post‐traumatic stress disorder.

*** *p* < 0.001.

^a^ adj.p, adjusted *p* value using beta values, adjusted for cell type, child age and sex, maternal age, maternal education, maternal marital status, maternal living area, prenatal smoking, and batch.

### 3.4 Candidate gene analysis

Seven HPA‐axis related candidate genes were examined, with a total of 407 CpGs sites (Table 4). After adjustment for multiple testing, 4.7% (*n* = 19) of these CpGs were significantly correlated with continuous cortisol levels. Correlation coefficients across these 19 CpGs ranged from *r* = −0.24 to +0.27. The strongest, and most significant correlation was that of *NR3C1* CpG cg14939152 with a correlation coefficient of *r* = 0.27, SE:0.08, H. Adj.*p* = 0.003.

**TABLE 4:** Candidate gene analysis of hypothalamic–pituitary–adrenal axis gene methylation and cortisol levels

Gene	Genomic region (hg19)	# EPIC probes^a^	Correlated CpGs^b^	CpG	Location (hg19)	r	H.adj.p
Brain derived neurotrophic factor (*BDNF)*	Chr11: 27670031–27747452	87	4	cg02386994	chr11:27723290	−0.20	0.029*
				cg27193031	chr11:27721088	0.26	0.004**
				cg25328597	chr11:27722638	0.21	0.022*
				cg04672351	chr11:27722889	0.23	0.012*
Corticotropin releasing hormone (*CRH)*	Chr8: 67088055–67091520	13	1	cg21240762	chr8:67089388	−0.23	0.011*
Corticotropin releasing hormone receptor 1 (*CRHR1)*	Chr17: 43853226–43926706	53	4	cg15834779	chr17:43919576	−0.18	0.046*
				cg10106856	chr17:43880210	0.20	0.028*
				cg03323388	chr17:43888776	−0.20	0.027*
				cg15844800	chr17:43916233	0.23	0.012*
Corticotropin releasing hormone receptor 2 (*CRHR2)*	Chr7: 30682632–30750930	47	2	cg26262196	chr7: 30696540	0.19	0.035*
				cg09797340	chr7:30749389	−0.23	0.012*
FK506‐binding proteins (FKBP) prolyl isomerase 5 (*FKBP5)*	Chr6: 35501361–35700207	57	1	cg26868354	chr6:35699952	0.20	0.026*
Nuclear receptor subfamily 3 group C member 1 (*NR3C1)*	Chr5: 142640230–142851250	85	3	cg14939152	chr5:142783831	0.27	0.003**
				cg21209684	chr5:142783848	−0.22	0.018*
				cg17349736	chr5:142802329	−0.25	0.007**
Nuclear receptor subfamily 3 group C member 1 (*NR3C2)*	Chr4: 148912932–149414546	67	4	cg09143276	chr4:149066518	−0.22	0.016*
				cg07335874	chr4:149136507	0.21	0.023*
				cg06240648	chr4:149289762	−0.19	0.039*
				cg23329208	chr4:149360981	0.23	0.011*
**Total**	**‐**	**407**	**19**				

Abbreviations: CpG, cytosine‐phosphate‐guanine; EPIC, Infinium MethylationEPIC array; H.adj.p, Holm multiple testing adjusted *p*‐value; hg19, Homo sapiens (human) genome assembly GRCh37; *r*, correlation coefficient.

* *p* < 0.05, ** *p* < 0.01.

^a^ total number of CpG probes in the gene region (after removal of cross reactive and failed probes).

^b^ probes with methylation significantly correlated with continuous cortisol levels after adjustment for multiple testing using the Holm method.

**FIGURE 2:**
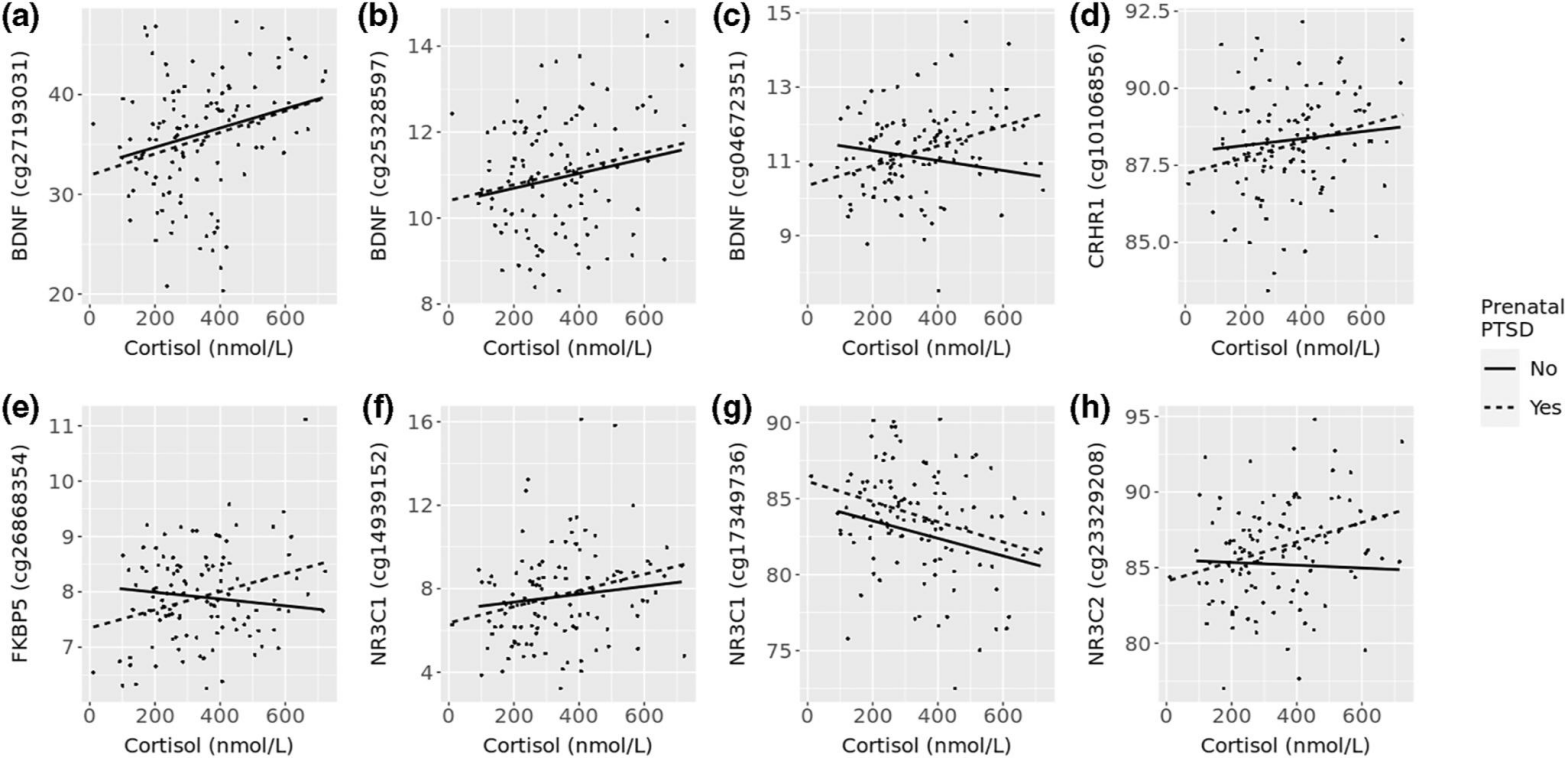
CpGs in hypothalamic–pituitary–adrenal HPA axis genes significantly correlated with cortisol levels in children whose mothers had prenatal post‐traumatic stress disorder (PTSD). (a) cg27193031 (*r* = 0.27, adj.*p* = 0.017), (b) cg25328597 (*r* = 0.2, adj.*p* = 0.034), (c) cg04672351 (*r* = 0.36, adj.*p* = 0.01), (d) cg10106856 (*r* = 0.24, adj.*p* = 0.009), (e) cg26868354 (*r* = 0.31, adj.*p* = 0.034), (f) cg14939152 (*r* = 0.26, adj.*p* = 0.033), (g) cg17349736 (*r* = −0.31, adj.*p* = 0.007), (h) cg23329208 (*r* = 0.33, adj.*p* = 0.019). CpGs first passed adjustment for multiple testing using Holm method *p* < 0.05. adj.p, *p* values after multivariate linear regression, adjusted for estimated blood cell proportions, child age and sex, maternal age, education, marital status, location and prenatal smoking status, and batch effects. CpGs shown are methylation measures which were significant in participant group with maternal PTSD. r = correlation coefficient in PTSD group. *Y*‐axis represents % methylation

In multivariate linear regression adjusting for confounding factors, methylation at seven of these CpGs remained significantly associated with cortisol levels (Table 5). Stratifying these findings by prenatal PTSD status, five remained significantly associated with cortisol levels in offspring with mothers with PTSD (Figure 2a,b,d,f,g), but none were associated with cortisol levels in the non‐maternal PTSD group. Interestingly, three significantly correlated CpGs which were not significant overall in the adjusted multivariate linear regression, were also found to be significant in the prenatal PTSD group (Table 5 and Figure 2 c,e,h).

**TABLE 5:** HPA‐axis cytosine‐phosphate‐guanine significantly correlated with continuous cortisol measures, stratified according to maternal post‐traumatic stress disorder

		Multivariate linear regression^a^	Stratified multivariate linear regression^a^	
		(*β* coef., 95%CI, adj.p)	(*β* coef., 95%CI, adj.p)	
Gene	CpG	*n* = 118	No prenatal PTSD (*n* = 33)	Prenatal PTSD (*n* = 85)
*BDNF*	cg27193031	0.008, −0.00003–0.016, 0.05*	−0.01, −0.10 to 0.08, 0.8	0.01, 0.002–0.02, 0.017*
	cg25328597	0.002, 0.0002–0.004, 0.03*	−0.0004, −0.009 to 0.007, 0.9	0.003, 0.0001–0.005, 0.034*
	cg04672351	0.001, −0.0003–0.003, 0.1	−0.002, −0.015 to 0.01, 0.7	0.002, 0.0006–0.004, 0.01*
*CRHR1*	cg10106856	0.002, 0.0001–0.005, 0.04*	−0.0008, −0.015 to 0.01, 0.9	0.004, 0.0009–0.006, 0.009**
	cg15844800	0.003, 0.0006–0.005, 0.01*	0.01, −0.008–0.03, 0.2	0.002, −0.0008–0.004, 0.2
*FKBP5*	cg26868354	0.0008, −0.0002–0.002, 0.1	−0.0004, −0.009 to 0.008, 0.9	0.002, 0.0001–0.003, 0.034*
*NR3C1*	cg14939152	0.003, 0.0004–0.007, 0.03*	0.001, −0.02–0.03, 0.9	0.004, 0.0003–0.008, 0.033*
	cg17349736	−0.008, −0.01 to −0.003, 0.001**	−0.02, −0.07 to 0.03, 0.4	−0.007, −0.013 to −0.002, 0.007**
*NR3C2*	cg07335874	0.002, 0.0004–0.003, 0.01*	0.002, −0.007–0.01, 0.6	0.001, −0.0003–0.003, 0.1
	cg23329208	0.004, −0.0007–0.008, 0.1	0.0009, −0.02–0.025, 0.9	0.006, 0.001–0.01, 0.019**

Abbreviations: *β* Coef. Regression coefficient; *BDNF*, Brain Derived Neurotrophic Factor; CpG, cytosine‐phosphate‐guanine; *CRHR1*, CRH Receptor 1; *FKBP5*, FKBP Prolyl Isomerase 5; H. adj.p, Holm multiple testing adjusted *p*‐value; *NR3C1*, Nuclear Receptor Subfamily 3 Group C Member 1; *NR3C2*, Nuclear Receptor Subfamily 3 Group C Member 1; PTSD, post‐traumatic stress disorder.

* p < 0.05, ** *p* < 0.01, *** *p* < 0.001.

^a^ using beta values, adjusted for cell type, child age and sex, maternal age, maternal education, maternal marital status, maternal living area, prenatal smoking and batch.

## 4 DISCUSSION

In this cohort of survivors of sexual violence during the Kosovo war (1998–1999), we have previously shown that maternal PTSD during pregnancy was associated with higher cortisol levels in the offspring, as well as differential methylation of HPA‐axis stress‐related genes (Hjort et al., 2021). We extend these findings in the current study, with the identification of sites across the epigenome where offspring DNA methylation was associated with cortisol levels. Furthermore, in stratified analyses, these associations were significant only in offspring born to mothers with PTSD during pregnancy. Together these findings suggest that PTSD during pregnancy plays a role in mediating aberrant cortisol signalling in offspring, in part regulated by DNA methylation.

There may be clinical utility in using DNA methylation markers of cortisol which reflect biological embedding of future mental health disease risk due to prenatal exposure (Aristizabal et al., 2020; Graham et al., 2019). DNA methylation measures could be used to measure risk of cortisol dysregulation and subsequent mental health issues, regardless of the knowledge of prenatal maternal mental health. They are also likely to reflect more stable changes in stress signalling and thus help explain long lasting associations with health outcomes occurring many years later (Nemoda & Szyf, 2017). In contrast, cortisol levels fluctuate over the day (Elder et al., 2016) and over time (Yiallouris et al., 2019) thus being less stable markers of future risk. Given the lack of prior studies which have investigated DNA methylation and cortisol levels, further studies are required to determine the true utility of these biomarkers. However, the fact that our observations were only seen in the PTSD group support the idea of transgenerational programing due to negative prenatal exposure.

There have been few previous epigenome‐wide association studies of cortisol levels. The first example of which found 22,425 sites associated with cortisol stress reactivity in a sample of 85 participants (Houtepen et al., 2016). None passed adjustment for multiple testing, so they focussed on three CpGs, cg27512205 (intronic region of *KITLG*), cg05608730 (upstream of *C1QTNF2*), and cg26179948 (intronic region of *JAZF1‐AS1*), which were also associated with childhood trauma, all of which were negatively correlated with cortisol. Another small study (*n* = 22) previously found early post‐conceptional maternal cortisol to be associated with multiple measures of methylation across the genome, with numbers varying in time points (between 2 and 1639 sites) (Barha et al., 2019). However, specific sites were not listed. Another more recent study of 318 participants, found one CpG, cg16290996 in the *GAS5* gene, was negatively correlated with morning cortisol levels (Lohoff et al., 2020). None of these CpGs were associated with cortisol levels in our study. Instead, we found that DNA methylation within three separate gene regions, as well as upstream of a separate gene, were most strongly associated with cortisol.

Methylation of CpG cg15321696, within the intronic region of the *FERM* *Domain Containing 4A* (*FRMD4A*) gene, cg00986889 in an exonal region of *Chromodomain Helicase DNA Binding Protein 4* gene, and cg15920527, approximately 44,500 base pairs upstream of the Coiled‐Coil Domain Containing 174 gene were negatively correlated with cortisol levels. No previous studies have investigated these genes in relation to cortisol, however differential methylation has been found in relation to diseases like Alzheimer's (Lambert et al., 2013; Yan et al., 2016), and for prenatal exposures like smoking (Breton et al., 2014; Küpers et al., 2015; Markunas et al., 2014; Richmond et al., 2018; Rzehak et al., 2016; S. Rauschert et al., 2019). A positive correlation was observed between methylation at cg18105800 within an exon of the *F‐Box And Leucine Rich Repeat Protein 2* gene, which encodes a subunit of a ubiquitin protein ligase complex, and cortisol (Matsushima et al., 2019). This is a novel finding.

Methylation at several genes involved in HPA‐axis regulation were associated with cortisol levels. After adjusting for multiple testing and covariates, seven CpGs were significantly correlated with cortisol levels, including cg27193031 and cg25328597 from *BDNF*, cg10106856 and cg15844800 from *CRHR1*, cg14939152 and cg17349736 from *NR3C1*, and cg07335874 from *NR3C2*.

One gene of particular interest *BDNF*, had three CpGs significantly correlated with cortisol after stratification. CpGs cg27193031 and cg04672351 are positioned in intronic regions either side of a CpG island overlapping the *BDNF* promotor region, where cg25328597 is positioned. The general dogma of DNA methylation is that methyl groups attached to DNA may block transcription, where the removal of these groups allow transcription, particularly in CpG islands (Dor & Cedar, 2018). Our observations suggest that *BDNF* may be dysregulated though DNA methylation mechanisms in response to transgenerational effects of PTSD.

BDNF is largely involved in neurobiological processes, such as dendritic growth, neurogenesis, and synaptogenesis and neuroplasticity (de Assis & Gasanov, 2019). BDNF is of interest to cortisol studies, as inhibition of glucocorticoid receptors indirectly disrupts BDNF signalling, which is implicated in issues with memory and development of mental illness. There have been many intergenerational animal studies, as well as a few human studies looking at maternal exposure as a modifier of offspring *BDNF* methylation, for example, studies of trauma and fear (Pilkay et al., 2020), bi‐polar disorder (Duffy et al., 2019), and depressive symptoms (Braithwaite et al., 2015). However, none of these observed *BDNF* methylation in the context of cortisol levels. When looking at specific CpGs, a separate study found that differential methylation (in placental tissue) at cg27193031 in offspring was significantly associated with maternal war related stress exposure in 24 mother/child dyads (Kertes et al., 2017). However, significance did not remain after adjustments. CpGs cg25328597 and cg04672351 seem to be novel observations. They have been reported (but not significant) in children, in relation to maltreatment in childhood (Weder et al., 2014) however, no previous studies reported their association with cortisol, or prenatal PTSD, in childhood or intergenerational studies.

Another gene of interest *NR3C1*, had two CpGs remain significant after stratification. Cytosine‐phosphate‐guanine dinucleotides cg17349736 which was negatively correlated with cortisol levels, is approximately 19,000 base pairs away from the *NR3C1* transcription site. Inversely cg14939152 was positively correlated with cortisol, and is within a CpG island covering the transcription site. Our contrast in findings suggest that aberrant patterns of methylation in the *NR3C1* promotor may affect *NR3C1* transcription. If transcription of *NR3C1* is blocked, then there will be less receptors to interact with available cortisol.

*NR3C1* encodes the glucocorticoid receptor. This receptor is a binding site for cortisol when it is released in response to acute and chronic stress (Gjerstad et al., 2018). One of the primary functions of the glucocorticoid receptor is to facilitate a negative feedback loop, halting the stress response, the process of which can been affected by sustained high levels of cortisol (Efstathopoulos et al., 2018). There are many intergenerational *NR3C1* DNA methylation studies of prenatal, perinatal, and early childhood exposure to maternal factors. These include but are not limited to, the effect of maternal care giving on infant methylation (Conradt et al., 2019), maternal support during stress (Bosmans et al., 2018), maternal psychosis (Palma‐Gudiel et al., 2015), and harsh parenting (Lewis et al., 2021). Interestingly, the harsh parenting study, including 97 children, also looked at *NR3C1* methylation and daily cortisol levels, and found that *NR3C1* methylation could predict a steeper daily cortisol slope (Lewis et al., 2021). However, methylation measures were not associated with morning, afternoon, or night cortisol levels. Our observations of specific CpG methylation of cg17349736 and cg14939152, being associated with cortisol levels are novel findings, particularly in relation to maternal PTSD status. Both cg17349736 and cg14939152 methylation in offspring have been studied in relation to maternal antenatal depression and anxiety (Bleker et al., 2019), however there was found to be no association. Only the one aforementioned study directly compared *NR3C1* methylation to cortisol measures (Lewis et al., 2021), and no studies have explored the relationship of cortisol and *NR3C1* methylation in response to maternal PTSD. In our study, after stratifying by prenatal PTSD status, all associations remained significant only in the PTSD group. Thus, our findings suggest there may be relationship between exposure in utero to adverse mental health and methylation of these particular CpGs.

### 4.1 Strengths and limitations

One of the strengths of this study was the collection of blood during a narrow two‐hour window in the morning. Collection time of cortisol is important as it is secreted diurnally, peaking approximately 1 hour after awakening (Nagamine et al., 2017). Thus, collecting measures at the same time for everyone is essential in cortisol studies. Further, this study used highly validated assessment tools, in the participant's native language to diagnose PTSD cases. To measure DNA methylation, we utilised one of the most commonly currently used arrays, the Illumina EPIC (Illumina Inc, 2020), which measures over 850,000 methylation sites. This allowed for both a hypothesis free analysis of the association between cortisol and methylation across the entire epigenome, as well as study of a priori selected, HPA‐axis candidate genes. Further, as the EPIC array is widely used, it will allow future researchers who use this platform to directly compare findings with our study. Because of the detailed characteristic data collected from participants, we were able to adjust for many factors that may have an influence over epigenetic differences such as age (C. P. Ryan, 2021), educational attainment (van Dongen et al., 2018), and maternal smoking during pregnancy (Sebastian Rauschert et al., 2019). The fact that many associations remained after adjusting for these factors strengthens the findings.

Our sample size is relatively small (*n* = 118) for an EWAS study which measures over 850,000 methylation sites. We took a less conservative approach in reporting findings after adjusting for multiple testing. They should therefore be interpreted with caution due to the increased risk of type I statistical errors (false positives) (Chen et al., 2017), and thus require independent replication in another study. The small sample size of this study also meant groups formed by stratification of maternal PTSD status were quite small, especially the control group (*n* = 33 compared to *n* = 85 for PTSD). This could have influenced the power to detect significant associations in that group. Finally, genetic factors could not be accounted for as maternal samples were not collected. Future studies should seek to include genotyping to assess genetic relatedness between mother and child. Further, although we exclude methylation at known SNPs, it is pertinent to include relevant SNPs within epigenetic analysis to assess whether methylation patterns may be driven by genetic variation.

## 5 CONCLUSION

To our knowledge, this is the first study to look at epigenome‐wide methylation in association with fasting cortisol levels in offspring and according to maternal prenatal PTSD. The relationship between DNA methylation and cortisol levels is largely understudied and considering it may have utility as a biologically embedded biomarker of future adverse mental health risk, this highlights an important unmet gap in research. This study has found that there is some level of evidence that differences in DNA methylation are associated with cortisol levels, and that this relationship is prominent in offspring whose mothers had prenatal PTSD. However, findings need to be replicated in larger cohorts that allow for greater statistical power to be confident of the observations of this study.

## CONFLICT OF INTEREST

This study was funded by the Foreign, Commonwealth and Development Office through the British Embassy in Pristina and the Danish Institute against Torture (Denmark). L. Hjort is funded by the Danish Diabetes Academy supported by the Novo Nordisk Foundation. J. Ryan is supported by a National Health and Medical Research Council Research Leader Fellowship (grant no.: 1135727). The contents of this publication are the sole responsibility of the authors and do not necessarily reflect the views of the Foreign, Commonwealth and Development Office. The authors have no other relevant affiliations or financial involvement with any organization or entity with a financial interest in or financial conflict with the subject matter or materials discussed in the manuscript apart from those disclosed.

## Supporting information

Supplementary Material 1

## Data Availability

Data are accessible from senior authors on reasonable request.
