# Supplementary material for: DNA methylation in blood cells is associated with cortisol levels in offspring of mothers who had prenatal post‐traumatic stress disorder
Source: Stress Health. 2022 Feb 10;38(4):755–66. doi: 10.1002/smi.3131 (PMC9790331; doi:10.1002/smi.3131)
Supplement: Supplementary file 1 — Supplementary Material 1 [file SMI-38-755-s001.docx]

Table S1. Probes removed at each step of quality control.

| **Type** | **Probes removed** |
| --- | --- |
| Failed probes (P>0.01) | 17,882 |
| Single Nucleotide polymorphisms | 177,445 |
| Sex chromosome | 17,080 |
| Cross reactive | 28,039 |
| Total remaining | **625,413*** |

*Initial number of probes before quality control =865,859

Table S2. Top differentially methylated CpGs associated with categorical cortisol levels, and PTSD stratification regression.

| **CpG** | **Location (hg19)** | **Gene** | **Cate Model^1^** | | **Stratified multivariate linear regression^2^ (Coef. 95%CI, adj.p)** | | |
| --- | --- | --- | --- | --- | --- | --- | --- |
|  |  |  | **Δ(%)** | **adj.p** | **No PTSD** | **Pregnancy PTSD** | |
| cg06941527^3^ | chr10:134775939 | *LINC01166* | +13.7 | 0.11 | NA | | 14.3, 7.81 – 20.85, **<0.001** |
| cg23987897^3^ | chr10:134775949 |  | +14.3 | 0.11 | NA | | 14.5, 7.74 – 21.20, **<0.001** |
| cg11043423^3^ | chr13:22341174 | > *FGF9* | +1.2 | 0.12 | NA | | 1.97, 0.97 – 2.94, **<0.001** |
| cg16863862^3^ | chr16: 69535862 | *CYB5B <* | -3.0 | 0.13 | NA | | -4.62, -7.17 – -2.07, **0.001** |
| cg07204800^4^ | chr2:242869193 | *LINC01237* | -1.9 | 0.13 | 0.48, -8.52 – 9.48, 0.89 | | -3.63, -5.82 – -1.45, **0.002** |
| cg26132462^4^ | chr7:4861712 | *RADIL* | -7.2 | 0.12 | -20.6, -47.9 – 6.67, 0.1 | | -12.2, -17.4 – -7.01, **<0.001** |

Δ, mean methylation difference (%) compared to ‘normal’ cortisol category; adj.p = adjusted p-value; CpG, cytosine-phosphate-guanine; *CYB5B*, Cytochrome B5 Type B, *FGF9*, Fibroblast Growth Factor 9; hg19, Homo sapiens (human) genome assembly GRCh37; *LINC01166*, Long Intergenic Non-Protein Coding RNA 1166; *LINC01237*, Long Intergenic Non-Protein Coding RNA 1237, r, correlation coefficient; *RADIL,* Rap Associating With DIL Domain.

> denotes that probe is downstream from the gene body/transcription site
< denotes that probe is upstream from the gene body/transcription site
^1^  using m values, adjusted for cell type, child age and sex, maternal age, maternal education, maternal marital status, maternal living area, prenatal smoking, batch, and Benjamini-Hochberg method for multiple testing.
^2^ using beta values, adjusted for same variables in Cate Model apart from multiple testing
^3^ associated with higher cortisol category compared to normal
^4^ associated with lower cortisol category compared to normal
NA sample size too small to run model.

Six separate CpGs were associated with categorical levels of cortisol after adjustments for multiple testing and controlling for estimated blood cell proportions, child’s age and sex, maternal age, education, marital status, living location and prenatal smoking status (p<0.15). These included four CpGs between normal and high cortisol, cg06941527 (Δ-13.7%, p=<0.001, BH.Adj.p=0.11), cg23987897 (Δ+14.3%, p=<0.001, BH.Adj.p=0.11), cg11043423 (Δ+1.2, p=0.003, BH.Adj.p=0.12) and cg16863862 (Δ+3.0, p=0.002, BH.Adj.p=0.13) and two CpGs significantly different between normal and low cortisol, cg07204800 (Δ-1.9%, p=0.001 BH.Adj.p=0.13), and cg26132462 (Δ-7.2%, p=<0.001, BH.Adj.p=0.12) . (Table S2, Figure S1, a-f). There were no CpGs with differential methylation associated with both high and low cortisol categories in comparison to the normal cortisol category after adjustment for multiple testing.

Figure S1. CpGs associated with categorical cortisol groups in epigenome-wide analysis


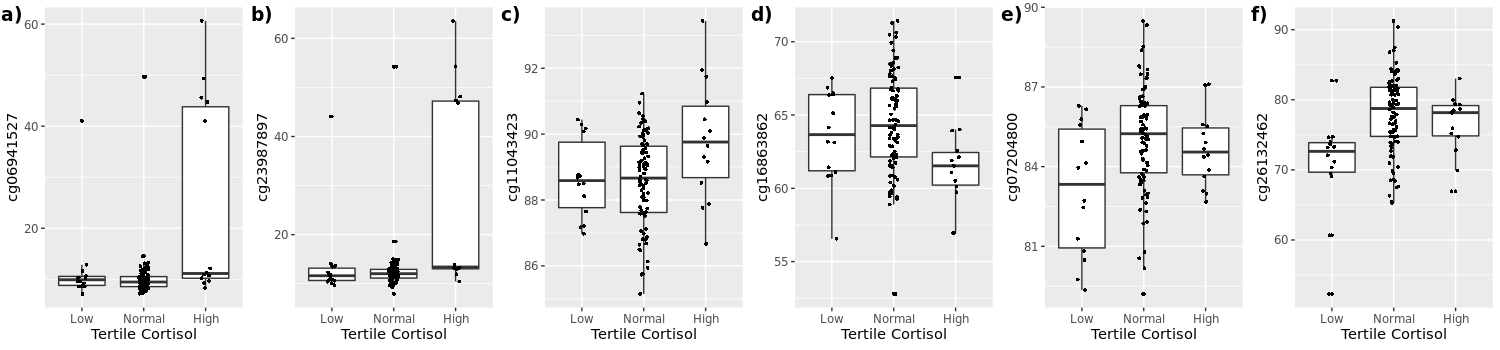


Plots a) to d) show differential methylation (difference of means) between normal cortisol levels (>171 to <549 nmol/L), and high cortisol levels (≥550 nmol/L). **a)** cg06941527 (Δ-13.7%, p=<0.001, BH.Adj.p=0.11), **b)** cg23987897 (Δ+14.3%, p=<0.001, BH.Adj.p=0.11), **c)** cg11043423 (Δ+1.2, p=0.003, BH.Adj.p=0.12) and **d)** cg16863862 (Δ+3.0, p=0.002, BH.Adj.p=0.13). Plots **e)** cg07204800 (Δ-1.9%, p=0.001 BH.Adj.p=0.13), and **f)** cg26132462 (Δ-7.2%, p=<0.001, BH.Adj.p=0.12), show differential methylation between normal cortisol levels (>171 to <549 nmol/L), and low cortisol levels (≤170 nmol/L).

BH.Adj.p= p value after adjustment for estimated blood cell proportions, child age and sex, maternal age, education, marital status, location and prenatal smoking status, batch effects, and Benjamini-Hochberg method multiple testing with a cut off of p<0.15. Y-axis represents % methylation. Whiskers represent 1.5x interquartile range from 75th and 25th percentile.
